# Corrigendum

**DOI:** 10.1111/jdi.13674

**Published:** 2021-10-11

The authors would like to draw the reader's attention to an error in the following article:

Seiichi T, Araki R, Motokura K, Tomobe Y, Yamauchi T, Hanaoka S, Tomotaki H, Iwanaga K, Niwa F, Takita J, Kawa M. Effects of passage through the digestive tract on incretin secretion: before and after birth. *J Diabetes Investig* 2021; 12: 970–977.

On page 971, the line “We used the χ^2^‐test to compare the proportions of categorical variables among the groups.” should be “Differences in the median values were compared using the Mann‐Whitney U‐test.”

The authors apologize for the errors and any inconvenience they may have caused.

